# Perspectives of Healthcare Providers and Managers on Older Adults’ Transition to a Long-Term Care Facility in the Côte-Nord Region of Quebec

**DOI:** 10.3390/geriatrics11040099

**Published:** 2026-08-06

**Authors:** Mélanie Le Berre, Fanny Leblanc, Mathieu Hotton, Marie-Pierre Gagnon, Félix Pageau, Émilie Dionne, Marie-Soleil Hardy, Maxime Sasseville, Samira Amil, Manon Cody, Louise Bertrand, André Côté, Maude Laberge

**Affiliations:** 1 Faculté de Médecine, Université Laval, Quebec, QC G1V 0A6, Canada; melanie.le-berre.1@ulaval.ca (M.L.B.); mathieu.hotton@fmed.ulaval.ca (M.H.); felix.pageau.1@ulaval.ca (F.P.); 2 VITAM Centre de Recherche en Santé Durable, Quebec, QC G1J 2G1, Canadamarie-pierre.gagnon@fsi.ulaval.ca (M.-P.G.); emilie.dionne3.ciussscn@ssss.gouv.qc.ca (É.D.); marie-soleil.hardy@fsi.ulaval.ca (M.-S.H.); maxime.sasseville@fsi.ulaval.ca (M.S.); samira.amil.1@ulaval.ca (S.A.); andre.cote@fsa.ulaval.ca (A.C.); 3 Centre de Recherche du CHU de Québec-Université Laval (CRCHUQ), Quebec, QC G1V 4G2, Canada; 4 Centre de Recherche du CISSS de Chaudière-Appalaches, Quebec, QC G6V 3Z1, Canada; 5 Centre Interdisciplinaire de Recherche en Réadaptation et Intégration Sociale (Cirris), Quebec, QC G1M 2S8, Canada; 6 Faculté des Sciences Infirmières, Université Laval, Quebec, QC G1V 0A6, Canada; 7 Faculté des Sciences Sociales, Université Laval, Quebec, QC G1V 0A6, Canada; 8 Centre Interuniversitaire de Recherche en Analyse des Organisations (CIRANO), Montreal, QC H3A 2M8, Canada; 9 Centre Intégré de Santé et de Services Sociaux de la Côte-Nord (CISSS-CN), Quebec, QC G5C 1P5, Canada; 10 Citizen Partner, Communauté Expériences, Unité de Soutien au Système de Santé Apprenant (SSA) Québec, Longueuil, QC J4K 0A8, Canada; 11 Faculté des Sciences de L’administration, Université Laval, Quebec, QC G1V 0A6, Canada

**Keywords:** long-term care, transitional care, aged, rural health services, life story

## Abstract

**Background/Objectives**: Older adults’ transitions to long-term care facilities (LTCFs) are complex. In Quebec, Canada, recent policies promoted LTCFs as living environments. The Life Story questionnaire was suggested as one tool to support this shift. In addition, rural regions may face specific barriers to successful transitions to LTCFs. The Côte-Nord region, with a vast territory and a low population density, offers a distinctly rural setting. The objective of this study was therefore to describe healthcare providers’ and managers’ perceptions of the use of the Life Story questionnaire during the transition to an LTCF in the Côte-Nord region of Quebec, and gain a deeper understanding of the factors influencing its implementation in this context. **Methods**: A qualitative study was conducted. Semi-structured interviews were audio-recorded, transcribed and analyzed using a descriptive interpretive methodology. **Results**: Participants highlighted social workers’ central role in completing the questionnaire and discussed its use by team members. They valued the tool for supporting individualized care and emotional well-being. They also identified barriers and facilitators at institutional, inter-institutional and organizational levels. Barriers included staff turnover and the difficulty of establishing a wide cultural shift. Facilitators included institutional support, consistent access to completed questionnaires, stable teams, interprofessional collaboration and inter-institutional collaboration. Participants finally offered some recommendations to spread the practice and foster a more consistent use across settings. **Conclusions**: The Life Story questionnaire appeared to support policy priorities and may be particularly valuable when completed prior to LTCF admission. Further research could explore its impact on specific indicators.

## 1. Introduction

The global population is aging [1], with rural areas experiencing some of the most significant demographic shifts [1,2]. As life expectancy increases, a growing proportion of the population may report multiple chronic conditions and comorbidities [3], which may increase the likelihood of requiring support with daily functioning. In some cases, this can lead to an admission to a long-term care facility (LTCF) [4]. In the United States, over 80% of LTCF residents are aged 65 years and older [5]. In Canada, older age has been associated with higher rates of LTCF admission [6], with more than 100,000 individuals aged 85 years and older residing in LTCFs in Canada in 2021. More specifically, in the Canadian province of Quebec, a recent report indicated that 91% of LTCF residents were aged 65 years or older [7]. As the population continues to age, the demand for long-term care services is expected to rise, highlighting the need for planning to ensure access to high-quality care for older adults.

While increasingly common, the transition from home to an LTCF remains challenging, notably due to the possibly complex health profiles of the newly admitted residents. Studies have reported risks of adverse outcomes during this period [8], such as increased behavioral and psychological symptoms of dementia or difficult behaviors, delirium, or adverse drug events [9,10]. Communication issues among professionals involved in the transition process, including physicians, nurses, social workers, and other allied healthcare professionals, could contribute to these risks. Missing, delayed or inaccurate clinical information such as care plans, prescriptions, or advance directives can result in fragmented or inadequate care [8]. In some cases, admissions to LTCFs can also occur rapidly and without prior planning, for instance following an acute health event, as many individuals and families may not have considered or discussed long-term care options in advance [11]. This can further complicate the transition process. In response to these difficulties, several transitional care initiatives targeting LTCFs have been developed [8,12].

Although LTCFs are integrated in the healthcare system, they also constitute residents’ living environments. However, the focus on medical care has sometimes overshadowed this role. In Quebec, Canada, a policy shift in 2003 formally redefined LTCFs not only as healthcare institutions, but also officially acknowledged them as living environments [13]. In line with person-centered care, the approach encouraged home-like settings and promoted flexible routines reflecting individual preferences [14]. In 2022, a new public policy and action plan further reinforced these values, recommending that care be tailored by recognizing each resident’s life story [7]. In this context, several tools have been introduced to understand residents’ backgrounds, identities, and preferences, including the Life Story questionnaire [15,16].

Life story work is an approach in which information about individuals, such as their history, interests, and preferences, is gathered and integrated into day-to-day care [17]. In several countries, life story work is recognized as a best practice and has been integrated into official healthcare policies [18,19]. It encompasses a broad set of tools and practices, such as a biographical summary or a care action plan, a personalized picture book, an audiotape or even a collage [7,17,20,21,22,23]. Among these tools, the Life Story questionnaire is a descriptive, non-standardized tool designed to capture personal details such as daily habits, meaningful relationships, and significant life events. While formats may vary across institutions, some LTCFs in Quebec, Canada, have begun incorporating the Life Story questionnaire during the admission process [24,25,26]. Depending on the resident’s capacity, the questionnaire may be completed independently, by a caregiver, or collaboratively. A translated example of a version used in the Côte-Nord region is provided in Appendix A.

While rural healthcare settings, including those in Quebec, face unique challenges and disparities in access and service utilization [27,28], research on the implementation of life story work initiatives in these settings remains limited. Rural regions may face specific barriers to successful transitions to LTCFs, including financial constraints, limited transportation options, greater workforce shortages, reduced bed availability in nearby facilities, and prolonged wait times [29]. The Côte-Nord region, with a vast territory and a sparse population density, offers a distinctly rural context for exploring these issues. The objective of this study is to describe healthcare providers’ and managers’ perceptions of the use of the Life Story questionnaire during the transition to an LTCF in the Côte-Nord region of Quebec, and gain a deeper understanding of the factors influencing its implementation in this context.

## 2. Methods

### 2.1. Study Design

This study was part of a broader research initiative examining innovations to improve healthcare trajectories from home to long-term care. The research team used a descriptive interpretive qualitative methodology, which provides a structured approach suited for investigating complex healthcare phenomena [30]. They adhered to the COnsolidated criteria for REporting Qualitative research (COREQ) for the planning and reporting of this study [31].

### 2.2. Selection of Participants

The research team recruited healthcare providers and managers from the local healthcare network serving the Côte-Nord region of Quebec, Canada, and from five of its LTCFs. Eligible participants were French-speaking adult employees of the local health network who had been involved in the residents’ admission process. A collaborator from the local health network (MC), who was also interviewed as a participant, shared the invitation to participate by email to the relevant facility of the local health network, identified eligible individuals and initiated contact. MC then provided contact information for the identified people to the research team. The research team contacted each person to provide further information about the project and to plan a time for an interview. Recruitment then followed a snowball sampling strategy. At the end of each interview, the interviewers asked the participant if they could think of other individuals who could be interested in participating. The research team then contacted the names provided by the participants. The collaborator did not have a direct supervisory relationship with the individuals contacted. A total of 16 individuals were personally contacted, including the initial participant and 15 individuals identified through snowball referrals. Fourteen healthcare providers and managers provided free and informed consent to participate in the interviews between Fall 2023 and Winter 2024, while two individuals declined participation. Data saturation was achieved through iterative analysis when successive interviews no longer generated new themes or subthemes, and previously identified patterns were consistently repeated across participants. Although data saturation informed recruitment decisions, it was not the sole criterion for ending participant enrollment and snowball sampling continued until no further referrals were received.

### 2.3. Data Collection

The research team conducted semi-structured interviews with healthcare providers and managers from the local health network, via videoconference using Microsoft Teams. The interviews were conducted by three members of the research team: a health services researcher (ML), a research associate with a background in social work and extensive experience working with older adults (FL), and a doctoral student in nursing science whose prior research has focused on older populations (MAB). Each interview was conducted by one to three of these researchers during the same videoconference session. ML, FL, and MAB participated in six, ten, and nine interviews, respectively. All three had previous experience in qualitative research. Having more than one interviewer present allowed one researcher to lead the interview while the other monitored the discussion, asked follow-up questions when appropriate, and managed logistical aspects of the videoconference. No differences in the depth or nature of the data were observed across interviews conducted under different interviewer configurations.

One participant had a prior professional relationship with the research team, having contributed to project ideation during three meetings held over several months before funding was secured, and subsequently remained involved as a member of the research team. All other participants had no prior relationship with any of the interviewers. The interviewers were familiar with the Life Story questionnaire and its intended use but approached the interviews with the aim of understanding participants’ experiences and perceptions rather than evaluating the intervention. The team developed an interview guide to support coherence and limit variability in how the interviews were conducted and the guide was used consistently across interviews. Before each interview, the interviewers explained the study objectives and procedures then followed a guide structuring the discussions. This guide was developed in collaboration with representatives of the local healthcare network to ensure the relevance and clarity of the questions. It comprised three main sections. The first section focused on the process of completing the Life Story questionnaire, with questions such as “In general, how and when is the Life story questionnaire completed?” or “Who is involved in completing the Life Story questionnaire?”. The second section addressed how the information collected through the questionnaire was used, with questions such as “How are the collected data from the questionnaire used and by whom? Could you provide an example of such a use?”. The third section explored the implementation of the new practice, sometimes involving the completion of the Life Story questionnaire prior to admission to the LTCF. This innovative practice had only been adopted in certain facilities within the Côte-Nord region. Questions in this section included “How did you implement the Life Story questionnaire, what were the barriers and facilitators?” and were adapted to the answers to the previous questions during the interview. A translation of the interview guide is provided in Appendix B. Interviews lasted between 17 and 68 min, with a mean duration of 39 min. Interviews were video-recorded and automatic transcripts were generated using Teams. A member of the research team reviewed the transcripts to ensure accuracy. The recordings and their transcripts were stored on a secure platform hosted on the University servers, with access restricted to members of the research team.

### 2.4. Data Analysis

The research team utilized NVivo 14 to analyze the interview transcripts through a hybrid deductive-inductive thematic approach [32], with initial themes derived from the three main sections of the interview guide, which had been co-developed with the local healthcare network. To mitigate the influence of the research team’s familiarity with the Life Story questionnaire and their professional backgrounds, coding and theme development were discussed among members of the research team to challenge individual interpretations, allowing assumptions and alternative interpretations to be questioned before reaching consensus. These discussions aimed to ensure that findings remained grounded in participants’ verbatims. Initially, two team members (FL, MAB) independently coded interview transcripts according to the three main themes derived from the interview guide. Any discrepancies were resolved through mutual consensus. Then, both conducted the first round of inductive thematic analysis on one interview and reconciled their results through consensus to refine the theme list. The team created matrices from this initial analysis to facilitate content extraction, and two team members (FL, MAB) independently coded another interview to ensure consistency and theme validity, resolving any discrepancies through consensus. This process aimed to enhance the rigor and trustworthiness of the analysis. Another round of coding was conducted with another interview to further validate the approach, with iterative discussions about the codes. After these tests, the matrices were finalized, and the team developed a preliminary code tree with definitions. The two team members then coded the remaining interviews. Finally, the team synthesized the content under each code in the code tree to complete the thematic analysis. The final code tree was presented to representatives of the local healthcare network to ensure coherence. A research member translated the most illustrative quotes (MLB).

## 3. Results

Most participants were women (*n* = 12/14, 86%) and represented 11 distinct professional roles, with a mean of 14 years of experience in their current professional role. Table 1 presents participant characteristics. Four main themes emerged from the interviews: *(1) the completion process*, which included subthemes related to the central role of social workers, the integration of the questionnaire into the preadmission process, and persistent gaps in continuity; *(2) its role in care delivery*, with subthemes addressing specific use for specific users, its integration from admission through long-term care use, and its contribution to individualized and compassionate care; *(3) the perceived benefits of using the questionnaire*, encompassing subthemes on humanizing care, adapting interactions and activities for more meaningful connections, and supporting emotional well-being; and (4) barriers and facilitators to completion and use, which addressed challenges such as staff shortage and turnover, and organizational change, contrasted with enabling factors like institutional support, access to the completed document, and final recommendations regarding this theme. Appendix C presents the summary of the themes, subthemes, and associated quotes.

### 3.1. Completing the Life Story Questionnaire

Participants indicated that social workers, either affiliated with the home care team or hospital-based, were most often responsible for completing the Life Story questionnaire, typically in collaboration with the seniors themselves, a family member, a caregiver, or a legal representative. Most participants reported that the questionnaire was completed during the preadmission process and was increasingly integrated into the early stages of registration for LTCF admission. They attributed this in part to recommendations from the Quebec Ministry of Health and Social Services. Some emphasized how their longstanding relationships with clients through home care facilitated earlier engagement in the process, sometimes beginning as soon as the decision to transition to a LTCF was confirmed: “*We know our clients well–sometimes we’ve followed them at home for many years. So we think we’re maybe the best person to fill it out with the family or the client, because we know them well.*” (PPAI-10). However, exceptions remained. Questionnaires were sometimes missing from transferred files, particularly when referrals came from other regions where preadmission completion was not standard practice. In such cases, LTCF staff completed the questionnaire upon admission. The time required to complete the questionnaire varied across settings and depended on institutional procedures and the availability of the individuals involved.

### 3.2. Using the Life Story Questionnaire in Care Delivery

The role of the Life Story questionnaire in care delivery varied depending on who was using it and which information they could access. For example, the personal support workers had access to a specific version, available in a dedicated large binder at their disposal. Support staff, such as those working in food services or housekeeping, were only informed of information directly relevant to their tasks and did not require access to the full content of the questionnaire. As a participant explained, “*It’s also about being mindful and showing some sensitivity. For example, if there’s a woman who’s experienced sexual violence, that’s something important for the PSWs and nurses to know, because they provide direct care. But it may not be necessary for the kitchen staff or the housekeeping team to know that*” (PPAI-02).

The use of the Life Story questionnaire began right at admission in some cases, and continued to inform care throughout the LTCF stay, becoming particularly useful as the resident’s memory declines or when caregivers are less available or no longer involved. As one participant reported, “*We can use the Life Story at any point, it’s not just for admission. It can be helpful in situations where the resident is having difficulty with hygiene care, or when we have questions, we’ll go check it, you know, it can be useful for any situation*.” (PPAI-01). The questionnaire supported efforts to provide individualized and compassionate care, helping staff tailor their approach to each resident’s background and preferences. One participant explained: “*If we’re providing care and the person becomes agitated, and we know they like flowers or a certain topic, then we talk about that. We’re way more likely to reach them than if we bring up something like baseball and they don’t care about it, you know? That’s why the Life Story is really essential to me.*” (PPAI-02).

### 3.3. Perceived Benefits of the Life Story Questionnaire

For the participants, the main benefit associated with the Life Story questionnaire, as previously noted, resided in its support of individualized and compassionate care. One explained how this humanized approach was reassuring for families during the preadmission process, “*Every time I prepared an admission, I’d sit down with the families to explain how things work at the facility and I’d ask them: ‘What did your father do in life? What did he enjoy? Did he volunteer?’ People were happy to hear those questions. They’d say, ‘I’m glad you’re asking that. You’re not just focused on physical needs, you also want to know who my parent was before their decline.’ So, for families, it made the care feel more human.*” (PPAI-02). Healthcare providers and managers also reported that the Life Story questionnaire facilitated their preparation for a new resident’s arrival and helped foster meaningful interactions. It was seen as helpful to guide targeted activities: “*We’re really able to gather a lot of information, which means we’re really able to better adapt our interventions with that person. If it’s someone who loves animals, for example, then we know that zootherapy could work well.*” (PPAI-09). The questionnaire was also seen as contributing to smoother care interventions by allowing staff to anticipate and respond more appropriately to residents’ behaviors or to navigate difficult situations. For example, a participant explained, “*Take the example of a man who used to work night shifts. If I’m a PSW and I show up at his door trying to force him back to bed, it’s going to cause a behavioral issue, anxiety, maybe disorganization. The nurse might have to give a [medication as needed to calm him]. It doesn’t work. She’ll call the doctor, and it turns into an escalation that we could avoid, for both the staff and the resident’s well-being.*” (PPAI-08). Finally, participants described the Life Story questionnaire as providing the teams with a sense of security, particularly in preparing to support newly admitted residents: “*Before the resident arrives, I’d say it reassures the staff. In fact, here, they even ask for it: ‘I haven’t seen the Life Story, is it at the nursing station?’ The personal support workers are curious to see who they’ll be working with.*” (PPAI-11).

### 3.4. Barriers and Facilitators to Implementation

Participants identified several barriers to the implementation and sustained use of the Life Story questionnaire in their practice, at the institutional, inter-institutional and organizational levels. They also shared some recommendations building on their observations.

#### 3.4.1. Institutional Level

At the institutional level, a commonly reported barrier was the persistent shortage and high turnover of staff, which hindered both the completion and integration of the tool into routine care. As one participant explained, “*Staffing challenges are real, there’s a shortage everywhere, and people come and go. Social workers already carry a heavy caseload, so finding the time to complete the questionnaire with residents is difficult. Many residents are alone and don’t have someone to complete it with them, which is also a barrier.*” (PPAI-13). Temporary personnel from private staffing agencies were seen as less familiar with the Life Story questionnaire: “*Our regular staff have developed the reflex of consulting the Life Story, but we often need to remind agency staff to do so: ‘Did you look at the Life Story?’ It’s not yet a reflex for them. Especially with temporary staff, the practice isn’t integrated—it’s not used everywhere in Quebec, so not everyone knows about it.*” (PPAI-11).

An important facilitator to the daily use of the Life Story questionnaire was ensuring easy access to its information. Some practical measures were viewed as particularly helpful: “*We make the Life Story questionnaires available at the nursing station before the resident arrives, and then we file them in the personal support workers’ binders under the resident’s tab. For example, in room 115, we insert the Life Story there for regular, daily consultation. They’re not buried in the file among archives. […] They’re also scanned into our shared drive in case we lose a paper copy.*” (PPAI-11). Support from the institution, stable teams, and well-established interprofessional collaboration were also seen as key to successful implementation.

#### 3.4.2. Inter-Institutional Collaboration

One participant emphasized the benefits of collaboration between home care and LTCF care, “*It’s the collaboration we have with home care staff. We used to be part of the same team, we had the same manager, so the practice emerged naturally. There’s trust between the caregiver and the home care worker, so it makes sense that the latter would be the one to gather that information. With the LTCF social worker, there’s no established relationship with the caregiver.*” (PPAI-08).

#### 3.4.3. Organizational Level

More broadly at the organizational level, the implementation of the questionnaire was seen as part of a wider cultural shift that required changing long-established work habits, an inherently difficult process. As one participant described, “*In theory, everyone would benefit from this, but even here in long-term care, it’s hard to change the culture. I could give you examples of changes I’ve been trying to make since I became a manager… There’s still resistance. People are used to doing things a certain way: ‘It’s working fine, why should I change?’ It’s hard to mobilize people when a change affects their routines and how they intervene.* (PPAI-02)”.

#### 3.4.4. Recommendations

Participants recommended standardizing the Life Story questionnaire process by formally assigning it to team members with appropriate training: “*I think it should be done by an educator, a social worker, or a human relations agent, a psychologist or someone like that. Someone with a background in intervention because sometimes powerful emotions come up. I’ve filled in Life Stories where the person started crying.*” (PPAI-02). Systematically engaging the caregivers directly and early in the process was also seen as beneficial, as one participant suggested, “*Ideally, we’d sit down with the family and complete the questionnaire together. If we just send it to them to fill out on their own, they often don’t really understand its purpose or what kind of information is important to include.*” (PPAI-02).

Finally, participants expressed a desire to spread the practice and ensure more consistent use across settings. Suggestions included raising awareness among staff who may be less familiar with the tool and implementing a standardized and concise format, such as a one-page summary. Making the information more accessible was also highlighted as important, as one participant noted, “*Just making it more visible would already be a big improvement. Maybe posting it in or near the resident’s room so that staff can easily say, ‘Oh right, this lady has this or that interest, maybe I can adapt how I interact.’ That would be better than having it tucked away in closed binders at the nursing station.*” (PPAI-07).

## 4. Discussion

This study described healthcare providers’ and managers’ perceptions of the use of the Life Story questionnaire in the transition to an LTCF in the Côte-Nord region of Quebec. Participants emphasized the central role of social workers in completing the questionnaire and discussed its specific use by different team members, from admission through ongoing care. The findings underscored the tool’s perceived value in supporting individualized and compassionate care by enabling personalized interactions and activities, fostering meaningful relationships, and promoting emotional well-being for both residents and care teams. Participants also identified several barriers and facilitators to its implementation, at the institutional, inter-institutional and organizational levels. Participants finally offered recommendations to spread the practice and foster a more consistent use across settings.

Consistent with the perceptions of healthcare providers and managers in the present study, previous research has highlighted the potential of life story work to enhance person-centered care in long-term care settings. A systematic review by Doran et al. (2019) [33] reported that life story work helped staff develop a deeper sense of knowing the resident as a person, with unique identities, preferences and life experiences. This greater understanding supported more individualized care, improved communication with residents and their families, strengthened therapeutic relationships, and promoted residents’ dignity. Previous studies have also suggested that adapting care based on residents’ life stories may contribute to fewer behavioral and psychological symptoms, including aggressive behaviors, and to improvements in the perceived quality of care [33,34,35,36]. These findings further support the potential of life story work tools, such as the Life Story questionnaire, to promote a more humanizing approach to care during the transition to long-term care.

A key benefit of the Life Story questionnaire identified was its potential to support continuity with the residents’ lives prior to admission by helping care teams tailor their interactions and activities based on personal histories. This sense of identity continuity with a past self has been recognized as critical to fostering the feeling of being “at home” in an LTCF, as highlighted in a grounded theory study [37]. These findings also reflect the intentions of Quebec’s 2003 policy shift [13] and its 2022 public policy and action plan [7], both of which emphasize the development of LTCFs as home-like environments. Ethnographic research similarly identified ‘continuing habits’ and ‘active emphasis on residents’ wishes’ as strategies to help seniors sustain continuity of self in these settings [38]. In this context, integrating the Life Story questionnaire appears well aligned with these policy priorities. Now implemented during the admission process in some LTCFs of the Côte-Nord region, the Life Story questionnaire could also be fostering continuity of care, another significant challenge during transitions to long-term care [39]. By contributing to informational continuity and supporting management continuity of residents’ needs, the questionnaire could be a valuable tool, particularly when introduced early in the process. From the interviews, this continuity was facilitated by inter-institutional collaboration between home care and LTCF care. The close connections between professionals, including familiarity through previous working relationships, may reflect characteristics of rural practice. Previous research has highlighted the strong sense of community often found in rural settings, where healthcare professionals are themselves members of the local community and frequently have longstanding knowledge of residents, their families, and local resources [40,41]. Such familiarity may facilitate transitions to long-term care by supporting informational continuity and collaboration across services. However, coordinating care remains challenging in rural settings because services are often geographically dispersed and rely on informal communication pathways that may be insufficient to ensure continuity of care [40]. Long travel distances and workforce shortages may further hinder collaboration [41].

Yet, participants also highlighted the importance of balancing accessibility of information with residents’ privacy and confidentiality. Sensitive information requires careful consideration regarding who should have access and under what conditions. Future implementation efforts should therefore establish clear processes regarding information sharing, including defining which information is necessary for specific roles, discussing residents’ preferences and consent regarding access to their personal information, and ensuring that increased visibility of the questionnaire does not compromise confidentiality. Previous work has suggested obtaining written consent when residents have the capacity to provide it, seeking assent from caregivers when appropriate, and, when neither is possible, relying on practices guided by the resident’s best interests and professional judgment [42].

Participants also identified staff turnover as a key barrier to completing and using the Life Story questionnaire. This challenge appears particularly acute in rural and northern communities of Canada [43]. Recruiting and retaining healthcare professionals in rural Canada remains difficult despite specific strategies attempting to address this issue, such as integrating rural experience in training or offering financial incentives [44,45,46]. High turnover may undermine relational continuity by limiting opportunities for residents and providers to develop stable, trusting relationships, an essential component of person-centered care [39]. Previous research has also linked high turnover to burnout in rural and northern Canada, due to adding on more work to account for short staffing, combined with an unspoken social contract leading to a heightened sense of personal responsibility for health outcomes within these smaller communities [47]. Yet, continuity in the individuals providing care has been shown to be particularly important to residents and may influence overall patterns of healthcare service use [48]. Moreover, frequent staff changes in LTCFs have been associated with lower quality of care and, in some studies, increased mortality [49,50]. These findings underscore the importance of staffing stability, particularly in rural settings, together with team-based implementation strategies to fully realize the potential benefits of tools like the Life Story questionnaire and to ensure high-quality care in long-term care settings.

A previous study on life story work similarly highlighted challenges in implementing these approaches despite their potential benefits, particularly because they can be time-consuming. In their systematic review, Doran et al. (2019) [33] emphasized the importance of organizational support and recommended a planned implementation approach that includes staff education and supervision. These implementation conditions are closely related to the staffing stability challenges identified in the present study, as sustained organizational support and staff engagement may be difficult to maintain in contexts characterized by frequent turnover. In addition, the timing of Life Story completion may influence implementation feasibility. A recent implementation study using a Life Story questionnaire reported higher completion rates when the questionnaire was introduced during the admission process compared with when it was introduced after residents had already moved into the facility [51].

### Limitations

This study had some limitations. First, recruitment was limited to five LTCFs, providing partial geographic coverage of the Côte-Nord region. Although this allowed for in-depth exploration, the findings may not reflect the full diversity of experiences across the region. Second, no participants from private staffing agencies were included. While participants did reference interactions with agency staff, their perspectives were conveyed indirectly, limiting the representation of agency workers’ experiences. However, as participants were permanent staff members integrated into their care teams, they possessed a deeper understanding of institutional processes and the practical use of the Life Story questionnaire, thus offering rich data. Lastly, as with all self-reported data, the findings may be influenced by social desirability bias, particularly given participants’ professional involvement in implementing the Life Story questionnaire. While the identified themes were presented to representatives of the local healthcare network to ensure coherence, no formal member checking or peer debriefing strategy was conducted. However, the convergence of themes across interviews supports the credibility of the findings.

## 5. Conclusions

This study described healthcare providers’ and managers’ perceptions of the Life Story questionnaire in the transition to long-term care in the Côte-Nord region of Quebec and the barriers and facilitators to its implementation in this context. Participants highlighted the role of social workers in completing the questionnaire and its use by various team members. The findings emphasized the tool’s value in supporting personalized care, fostering meaningful relationships, and promoting emotional well-being. However, barriers such as staff turnover and the difficulty of establishing a wide cultural shift were identified. Facilitators included institutional support, consistent access to completed questionnaires, stable teams, interprofessional collaboration and inter-institutional collaboration. Recommendations were made to improve the tool’s widespread and consistent use in the Côte-Nord region of Quebec. Further research could examine more specific application of the tool and its impact on other indicators, potentially through chart review and explore its implementation in other regions of the province.

## Figures and Tables

**Table 1:** Characteristics of healthcare providers and managers participants (*n* = 14).

Gender	Professional Role	Work Setting	Years of Experience in Their Professional Role
Man	Special care counsellor	Long-term care facility	25
Man	Long-term care facility manager	Long-term care facility	2
Woman	Long-term care facility manager	Long-term care facility	21
Woman	Long-term care facility manager	Long-term care facility	5
Woman	Social worker	Long-term care facility	16
Woman	Clinical activities specialist/Social worker	Home care team	28
Woman	Executive counsellor	Direction of the Support program for the autonomy of seniors	20
Woman	Executive counsellor	Direction of the Support program for the autonomy of seniors	3
Woman	Assistant head nurse	Long-term care facility	17
Woman	Physiotherapy technologist	Long-term care facility	8
Woman	Human relations agent	Home care team	20
Woman	Personal support worker	Long-term care facility	15
Woman	Unit head nurse	Long-term care facility	3
Woman	Licensed practical nurse	Long-term care facility	8

## Data Availability

The data presented in this study are available upon reasonable request from the corresponding author due to privacy reasons.

## Abbreviations

The following abbreviations are used in this manuscript:

LTCF	Long-term care facility
COREQ	COnsolidated criteria for REporting Qualitative
OT	Occupational therapist

## Appendix A. Translated Example of the Life Story Questionnaire

Last Name: _____________________   First Name: _____________________________

How would the resident like to be addressed: _________________________________

Previous living situation: ☐ Home ☐ Apartment ☐ Other: _______________________

Spoken language: ☐ French ☐ English ☐ Other: ________________________________

Civil status: ☐ Single ☐ Widow ☐ Separated

☐ Married/Common-Law   Since: _________   Name of partner: ________________

Does the resident recognize their partner: ☐ Yes ☐ No

Place of birth: ________________________________

Significant living place from 18 years old to today: ______________________________

Owned: ☐ House ☐ Cottage/Chalet ☐ Apartment building ☐ Business

Favorite room of the house: ______________________

Main profession: _______________  Main place of work: _______________________                      ☐ Day Shift ☐ Evening Shift ☐ Night Shift

Education level: _______________________________

Religion: ____________________________________

Believing: ☐ Yes   ☐ No     Practicing: ☐ Yes   ☐ No

⁡

The resident had children: ☐ Yes   ☐ No      Number of children: __________________

⁡

Name and order of children	Alive	Sex	Recognized by the resident	Relationship with the resident
	☐ Y ☐ N	☐ M ☐ F	☐ Y ☐ N	
	☐ Y ☐ N	☐ M ☐ F	☐ Y ☐ N	
	☐ Y ☐ N	☐ M ☐ F	☐ Y ☐ N	
	☐ Y ☐ N	☐ M ☐ F	☐ Y ☐ N	

⁡

☐ Mandated person                 Name: ___________________

☐ or legal representative               Contact details: _____________

⁡

Significant relationships (friends, extended family, neighbors…)		
Name	Relationship	Visits the resident
		☐ Y ☐ N
		☐ Y ☐ N
		☐ Y ☐ N

⁡

Specific life circumstances:

	0–20 years old	20–40 years old	40–60 years old	After 60 years old
Best memories				
Emotionally significant events				

⁡

Personality:

Among the following traits, indicate those that correspond to the resident’s personality over the course of their life:

☐ Extrovert ☐ Introvert ☐ Serious ☐ Calm ☐ Nervous/Anxious ☐ Shy ☐ Cheerful

Other traits or description of the resident’s personality: ________________________

__________________________________________________________________________

__________________________________________________________________________

Changes in personality since loss of autonomy: _________________________________

__________________________________________________________________________

__________________________________________________________________________

Fears or beliefs since loss of autonomy: _______________________________________

__________________________________________________________________________

Dislikes of the resident (ex. being touched, topics to avoid, etc…): ________________

__________________________________________________________________________

__________________________________________________________________________

Emotional challenges or particular behaviors and coping mechanisms: ___________

__________________________________________________________________________

__________________________________________________________________________

⁡

⁡

Previous life habits

Eating behaviors

Breakfast: Time of day: _______________    Duration: ___________________________

Lunch: Time of day: _________________    Duration: ____________________________

Supper: Time of day: ________________    Duration: ____________________________

Importance attached to breakfast: ____________________________________________

Frequent to skip a meal: ☐ Yes    ☐ No

Snacks during the day: ☐ Yes  ☐ No   Time of day: _______________________                              Types of snacks: _____________________

Other eating behaviors: _____________________________________________________

Hydration

Hot beverages: ________________  Cold beverages: ____________________________

Time of day: _______________________

Hygiene

☐ Bath   ☐ Shower   ☐ Sink    Time of day: ___________________________________

☐ Took care of appearance   ☐ Disliked to wash    ☐ Sensitive to cold

Body and beauty care: ______________________________________________________

Clothing

Favorite clothing: __________________________________________________________

Specific habits (ex. enjoys getting dressed at a specific time of day, etc…): _________

__________________________________________________________________________

⁡

⁡

Activities, leisure, pastime, source of stimulation

Entertainment and social activities (ex. playing cards (which game?), cooking, bingo, watching television (which programs?), etc…)	Frequency
	
	
	
	
	

⁡

The resident listens to music: ☐ Y   ☐ N   Music style: ___________   Frequency: ___

Likes to dance: ☐ Y   ☐ N   Style: ____________   Frequency: ____________________

Likes to sing: ☐ Y   ☐ N

Spiritual or religious activities: ☐ Y   ☐ N    Precisions: _________________________

⁡

Intellectual and creative activities (ex. reading books, newspapers, crosswords, sewing, knitting, puzzles, board games (which ones?), etc…)	Frequency
	
	
	
	

⁡

Physical or outdoor activities (ex. walking, fishing, cycling, hunting, golfing, skiing, etc…)	Frequency
	
	
	
	
	

⁡

Skill games (ex. darts, bowling, pool, pétanque, etc…)	Frequency
	
	
	
	

⁡

Additional relevant information: _____________________________________________

__________________________________________________________________________

__________________________________________________________________________

⁡

Confidentiality

I authorize the healthcare providers taking care of ______________________________ to access the information contained in this form. I agree that the information will be made available to all parties involved in the care of my relative.

⁡

____________________________________                 __________________

Signature of the resident or the caregiver                      Date

⁡

_____________________________________

Signature of the healthcare worker (if present)

## Appendix B. Translated Interview Guide

Pre-Interview Checklist

☐ Read the project presentation and obtain verbal consent from the participant.
☐ Answer any questions regarding data use and confidentiality.
☐ Explain that the interview will be recorded.
☐ Present the interview context.

Interview Context

Hello, my name is [Name], and I am [Role] on the project team. Thank you for taking the time to participate in this interview, which should last between 30 and 45 min.

The purpose of this interview is to better understand the transition process of older adults into long-term care facilities (CHSLDs), and more specifically, to explore a practice implemented in certain CHSLDs within the CISSS de la Côte-Nord (CISSS-CN) regarding the use of the Life Story questionnaire.

During this interview, I will ask you questions about:

- Your roles and responsibilities as a [participant’s role] and how you collaborate with other team members;
- Factors that facilitate or hinder the admission and transition process;
- The impact of geographical context on this process.

Please feel free to skip any question you are not comfortable answering. The interview will be recorded, but you may request to stop the recording at any time.

Interview Start

Consent Question:

Do you agree to this interview being recorded and to the data being used anonymously for research and quality improvement purposes?

Let’s begin with a few questions about your professional background.

Section 1: Participant Background

1. Can you tell us about yourself?
  ○ What is your job title?
  ○ How long have you been in your current position?
  ○ How many years of experience do you have?

Section 2: The Life Story questionnaire

2. Regarding the Life Story process:
  ○ If interviewing a social worker or nurse:
    ▪ How is the Life Story questionnaire completed?
    ▪ Who is involved in completing it?
    ▪ When is it typically completed?
  ○ If interviewing another type of staff member or manager:
    ▪ What do you know about the Life Story questionnaire?
    ▪ In your opinion, who is involved in completing it?
    ▪ When is it usually completed?

We understand that the Life Story questionnaire is generally completed upon the resident’s arrival, although some teams complete it beforehand.

3. On early completion of the Life Story questionnaire:
  ○ If interviewing a social worker or nurse:
    ▪ Why is it completed before the admission?
    ▪ What motivated this approach?
    ▪ In what context is it done?
  ○ If interviewing another staff member or manager:
    ▪ In your opinion, why is it completed in advance?

4. What benefits do you see from this practice?
  ○ For staff?
  ○ For residents and their caregivers?

5. How was this practice implemented?
  ○ What were the facilitating factors?
  ○ What challenges were encountered?
  ○ What was the broader context?

6. Do you think that completing the Life Story questionnaire before the resident’s arrival should become standard practice?
  ○ Why?
  ○ What might make this difficult to implement?

7. How is the information from the Life Story questionnaire used?
  ○ Who has access to it?
  ○ Who reads it?
  ○ Can you share an example of how information from the Life Story questionnaire has been used in practice?

8. What could be done to better integrate the Life Story questionnaire into practice?
  ○ To improve resident admission?
  ○ To personalize their new living environment?

Section 3: Transition Process

9. In your opinion, what are the key steps in the transition to a CHSLD?
10. Would you be willing to map out the service pathway of an older adult - from before admission to their move into the CHSLD - and email it to us following the interview?

Conclusion

11. Is there anything we haven’t covered that you would like to discuss?
12. Could you refer us to other individuals you believe would be relevant to interview for this project?

Follow-Up Permission

If we need clarifications on any of your responses, would you agree to be contacted by email?

Thank you again for taking the time to speak with us.

## Appendix C. Summary of the Themes, Subthemes, and Associated Quotes

**Theme**	**Subtheme**	**Relevant Illustrative Quote**
Completing the Life Story questionnaire	Central role of social workers	“There are families who are very capable of completing it on their own, and they do. The social worker, in theory—well, in best practice, and that’s how we try to do it here—reviews the Life Story to make sure there’s no missing information, or helps the family and/or the person complete it.” (PPAI-06) “Usually, the social worker fills it out either with the person or with a representative–either a relative or a legal guardian.” (PPAI-07) “Less commonly, it’s the hospital social workers. If the person was admitted to hospital and didn’t have a social worker before, then it’s the hospital team that provides [the information] to us.” (PPAI-09)
	Integration into the preadmission process	“We explain why it’s important and what kind of information we need in the Life Story–what will help us provide care, and better quality care.” (PPAI-02) “We’re seeing fewer and fewer Life Story questionnaires left incomplete before admission. Over the past year, there’s been real improvement–especially thanks to the Ministry’s recommendations. Before that, it was more difficult.” (PPAI-07) “We know our clients well–sometimes we’ve followed them at home for many years. So we think we’re maybe the best person to fill it out with the family or the client, because we know them well.” (PPAI-10)
	Exceptions and gaps in continuity	“When the request comes from outside the region, I’ll be the one to complete it with the family.” (PPAI-01)
Using the Life Story questionnaire in care delivery	Specific use for specific users	“Currently, there’s a copy of the Life Story—the original is kept in the resident’s file. That file is accessible to nurses, licensed practical nurses, doctors, social workers, physiotherapists, and occupational therapists (OT). For us, physio and OT are important, and they’ll refer to the Life Story when a situation arises. So, they all have access to the Life Story through the medical file on the unit. We also make a copy to include in the binder used by the personal support workers (PSWs). So the PSWs have access, and we also make a copy for the recreation staff, they have a binder with the Life Stories and they can consult it too.” (PPAI-05) “It’s also about being mindful and showing some sensitivity. For example, if there’s a woman who’s experienced sexual violence, that’s something important for the PSWs and nurses to know, because they provide direct care. But it may not be necessary for the kitchen staff or the housekeeping team to know that, you know?” (PPAI-02)
	From admission through long-term care	“At the team level, when they’re admitting someone, we definitely have a debriefing or team meeting where we discuss that a new resident is coming in, what they like, if they stay up late, and we’ll cover the basics. Then, we quickly make the Life Story accessible so they can refer to it, especially for things like the person’s habits, so they don’t have to wait a week wondering if they need a little light at night to sleep, it’s something they’ll already know.” (PPAI-01) “We can use the Life Story at any point, it’s not just for admission. It can be helpful in situations where the resident is having difficulty with hygiene care, or when we have questions, we’ll go check it, you know, it can be useful for any situation.” (PPAI-01)
	Contribution to individualized and compassionate care	“If we’re providing care and the person becomes agitated, and we know they like flowers or a certain topic, then we talk about that. We’re way more likely to reach them than if we bring up something like baseball and they don’t care about it, you know? That’s why the Life Story is really essential to me.” (PPAI-02) “If a resident tells us, ‘I need to get to work,’ we can meet them in their reality by saying something like, ‘Well, today’s Sunday, I think you might have the day off.” (PPAI-12)
Perceived benefits of the Life Story questionnaire	Humanizing care	“Every time I prepared an admission, I’d sit down with the families to explain how things work at the facility and I’d ask them: ‘What did your father do in life? What did he enjoy? Did he volunteer?’ People were happy to hear those questions. They’d say, ‘I’m glad you’re asking that. You’re not just focused on physical needs, you also want to know who my parent was before they lost so much of their independence.’ So, for families, it made the care feel more human.” (PPAI-02)
	Adapting interactions and activities for more meaningful connections	“The Life Story teaches us a lot about the resident’s past. It helps us build a positive relationship, especially with people who aren’t able to express themselves much anymore. If we know their story, whether they have children, or not at all, what their previous job was… It opens up opportunities for conversation when they arrive. We can ask questions and try to connect. It really makes things easier for staff.” (PPAI-11) “We’re really able to gather a lot of information, which means we’re really able to better adapt our interventions with that person. If it’s someone who loves animals, for example, then we know that zootherapy could work well.” (PPAI-09)
	Supporting emotional well-being	“Take the example of a man who used to work night shifts. If I’m a PSW and I show up at his door trying to force him back to bed, it’s going to cause a behavioral issue, anxiety, maybe disorganization. The nurse might have to give a PRN. It doesn’t work. She’ll call the doctor, and it turns into an escalation that we could avoid, for both the staff and the resident’s well-being.” (PPAI-08) “Before the resident arrives, I’d say it reassures the staff. In fact, here, they even ask for it: ‘I haven’t seen the Life Story, is it at the nursing station?’ The personal support workers are curious to see who they’ll be working with.” (PPAI-11)
Barriers and facilitators to completion and use of the Life Story questionnaire	Institutional level: Staff shortage and turnover	“Staffing challenges are real, there’s a shortage everywhere, and people come and go. Social workers already carry a heavy caseload, so finding the time to complete the questionnaire with residents is difficult. Many residents are alone and don’t have someone to complete it with them, which is also a barrier.” (PPAI-13) “Our regular staff have developed the reflex to consult the Life Story, but we often need to remind agency staff to do so: ‘Did you look at the Life Story?’ It’s not yet a reflex for them. Especially with temporary staff, the practice isn’t integrated—it’s not used everywhere in Quebec, so not everyone knows about it.” (PPAI-11)
	Institutional level: Access to the information	“We make the Life Story questionnaires available at the nursing station before the resident arrives, and then we file them in the personal support workers’ binders under the resident’s tab. For example, in room 115, we insert the Life Story there for regular, daily consultation. They’re not buried in the file among archives. […] They’re also scanned into our shared drive in case we lose a paper copy.” (PPAI-11)
	Inter-institutional collaboration: Collaboration between home care and LTCF care	“It’s the collaboration we have with home care staff. We used to be part of the same team, we had the same manager, so the practice emerged naturally. There’s trust between the caregiver and the home care worker, so it makes sense that the latter would be the one to gather that information. With the LTCF social worker, there’s no established relationship with the caregiver.” (PPAI-08)
	Organizational level: Cultural shift	“In theory, everyone would benefit from this, but even here in long-term care, it’s hard to change the culture. I could give you examples of changes I’ve been trying to make since I became a manager… There’s still resistance. People are used to doing things a certain way: ‘It’s working fine, why should I change?’ It’s hard to mobilize people when a change affects their routines and how they intervene. (PPAI-02)
	Recommendations	“I think it should be done by an educator, a social worker, or a human relations agent, a psychologist or someone like that. Someone with a background in intervention because sometimes powerful emotions come up. I’ve filled in Life Stories where the person started crying.” (PPAI-02) “Ideally, we’d sit down with the family and complete the questionnaire together. If we just send it to them to fill out on their own, they often don’t really understand its purpose or what kind of information is important to include.” (PPAI-02) “Just making it more visible would already be a big improvement. Maybe posting it in or near the resident’s room so that staff can easily say, ‘Oh right, this lady has this or that interest, maybe I can adapt how I interact.’ That would be better than having it tucked away in closed binders at the nursing station.” (PPAI-07)
